# Traditional Chinese Medicine Syndrome Differentiation of Adult Patients With Type 2 Diabetes and Metabolic Syndrome: Protocol for a Cross-Sectional Study

**DOI:** 10.2196/86217

**Published:** 2026-01-23

**Authors:** Jialing Zhang, Hoi Ki Wong, Zhilin Lin, Shuyan Zhong, Minxia Ma, Xuejiao Wang

**Affiliations:** 1 Vincent V.C. Woo Chinese Medicine Clinical Research Institute School of Chinese Medicine Hong Kong Baptist University Hong Kong SAR China (Hong Kong); 2 School of Chinese Medicine Hong Kong Baptist University Hong Kong SAR China (Hong Kong); 3 Children's Diagnosis and Treatment Center of the Affiliated Hospital Changchun University of Chinese Medicine Changchun, Jilin China

**Keywords:** Traditional Chinese Medicine, syndrome differentiation, type 2 diabetes, metabolic syndrome, personalized treatment

## Abstract

**Background:**

The global burden of type 2 diabetes mellitus (T2DM) and metabolic syndrome (MetS) continues to rise, with these conditions significantly increasing risks of cardiovascular disease, disability, and mortality. Traditional Chinese Medicine (TCM) syndrome differentiation, a cornerstone of TCM practice, guides diagnosis and treatment by identifying patterns of disharmony. However, large-scale studies investigating TCM syndrome patterns in T2DM comorbid with MetS remain scarce.

**Objective:**

This cross-sectional study aims to characterize TCM syndrome profiles in a population diagnosed with T2DM and MetS and evaluate their diagnostic relevance.

**Methods:**

This cross-sectional study will enroll a cohort of 470 participants diagnosed with T2DM and MetS. All participants will undergo comprehensive assessments, including the Syndrome Differentiation Questionnaire for T2DM and MetS, demographic and anthropometric measurements, biochemical profiling (eg, fasting glucose, glycosylated hemoglobin, and lipid panel), dietary measurement (Food Frequency Questionnaire), physical activity measurement (International Physical Activity Questionnaire Short Form), sleep quality evaluation (Pittsburgh Sleep Quality Index), quality-of-life assessment (Audit of Diabetes-Dependent Quality of Life), stroke risk estimation (Framingham Stroke Risk Score), and retinal imaging. Latent class analysis will be used to identify the TCM syndrome patterns. Factor analysis will be employed to identify core TCM syndrome factors. Hierarchical cluster analysis will be performed to classify TCM syndrome elements, and logistic regression will examine associations between syndrome differentiation, metabolic parameters, lifestyle factors, and disease progression.

**Results:**

This trial was registered on November 17, 2024. Participant recruitment for this study was initiated in November 2024. As of October 2025, more than 450 eligible participants have been enrolled and have completed data collection. Recruitment is scheduled to conclude on December 31, 2025.

**Conclusions:**

As the first large-scale clinical study to systematically characterize TCM syndrome differentiation in T2DM-MetS comorbidity, this research will establish syndrome profiles associated with metabolic parameters, lifestyle factors, and disease progression. The findings are expected to provide a framework for integrating TCM syndrome differentiation into chronic disease management, ultimately contributing to personalized treatment strategies and improved patient outcomes in integrative medicine.

**Trial Registration:**

ClinicalTrials.gov NCT06703684; https://clinicaltrials.gov/study/NCT06703684

**International Registered Report Identifier (IRRID):**

DERR1-10.2196/86217

## Introduction

Diabetes mellitus (DM) presents a critical global health challenge, with its prevalence escalating rapidly. As of 2021, approximately 537 million adults were affected, with projections estimating a rise to 783 million by 2045 without effective preventive measures [1]. Type 2 diabetes mellitus (T2DM) constitutes over 90% of these cases, underscoring its significance in the diabetes epidemic [2]. In Hong Kong, T2DM impacts nearly 10% of the adult population, mirroring global trends [3]. Adults with DM exhibit a 2-4 times higher risk of cardiovascular complications compared to adults with no DM [4]. Alarmingly, 65%-85% of patients with T2DM meet the diagnostic criteria for metabolic syndrome (MetS) [5,6], characterized by abdominal obesity, hypertension, dysglycemia, and dyslipidemia [7,8]. MetS impacts 20%-25% of the global population [9] and serves as an independent predictor of mortality among individuals with diabetes. This coexistence exacerbates metabolic abnormalities and increases cardiovascular risk, underscoring the imperative for early diagnosis and intervention to enhance management and prevent chronic complications [10,11].

Although conventional pharmacotherapy targets individual MetS components (eg, hypoglycemics, statins, and antihypertensives), emerging evidence supports Traditional Chinese Medicine (TCM) as a complementary approach. Clinical trials demonstrate that integrating TCM with Western regimens yields superior improvements in anthropometrics (eg, waist/hip circumference), glycemic control (glucose, glycosylated hemoglobin), insulin resistance index, and triglycerides, with minimal adverse reactions [12]. This aligns with a systematic review of 16 randomized controlled trials, where TCM significantly enhanced weight management and metabolic regulation [13]. Mechanistically, TCM compounds modulate lipid metabolism, vascular endothelial function, and insulin sensitivity via the phosphatidylinositol 3-kinase/protein kinase B signaling pathway, peroxisome proliferator-activated receptor pathway, and AMP-activated protein kinase pathway, offering multitargeted correction of metabolic dysregulation [14]. Together, clinical and pharmacological evidence supports TCM’s role in addressing the complex interplay of T2DM and MetS.

TCM practice relies on accurate diagnosis and treatment procedures known as Bian Zheng Lun Zhi (syndrome differentiation followed by treatment procedures). This vital concept involves a comprehensive analysis of clinical information obtained through 4 main diagnostic methods: observation, listening, questioning, and pulse analysis. The integrated data then guides the selection of TCM therapeutic interventions such as acupuncture and herbal formula prescriptions [15]. For DM, different syndromes exhibit distinct metabolic characteristics and corresponding clinical biomarkers, thereby guiding TCM therapies in alignment with this principle [16]. However, consensus on syndrome differentiation among practitioners is often hindered by a lack of standardized terminology [17], and research on syndrome differentiation for T2DM comorbid with MetS remains limited. This study aims to identify prevalent TCM syndromes in individuals with T2DM and MetS. Additionally, it will examine how these syndromes are associated with health-related behaviors (eg, physical activity, diet, sleep) and quality of life. By identifying specific TCM syndrome patterns that will correlate with particular metabolic abnormalities and varying risks of disease progression, this study may enhance diagnosis and guide personalized treatment strategies.

## Methods

### Study Design

We present a protocol for a cross-sectional study. This study will be conducted at the Chinese Medicine Clinics of Hong Kong Baptist University in Hong Kong Special Administrative Region, China. Data collection will be conducted over a 12-14 month period, commencing in November 2024.

### Ethical Considerations

This study has been approved by the Research Ethics Committee of Hong Kong Baptist University (REC/23-24/0564) and has been registered at ClinicalTrials.gov (NCT06703684). Volunteers will be required to provide written informed consent. The reporting of this study follows the recommendations of the STROBE (Strengthening the Reporting of Observational Studies in Epidemiology) guidelines [18].

### Study Procedure and Population

We aim to recruit a cohort of 470 adult participants (age ≥18 years) diagnosed with both T2DM and MetS [19]. The inclusion and exclusion criteria are detailed in Textbox 1. Following screening, enrolled participants will proceed to complete the study assessments. Participants who complete all study procedures will receive a transportation allowance of HK$100.

**Inclusion and exclusion criteria for participation.**

**Inclusion criteria**

- Age between 18 and 75 years
- Diagnosed with type 2 diabetes mellitus
- Diagnosed with metabolic syndrome according to guidelines for the prevention and treatment of type 2 diabetes mellitus in China (2020 edition) from Chinese Diabetes Society. People who meet 3 of the following diagnostic criteria or more can be diagnosed with metabolic syndrome [19]: (1) abdominal obesity, that is, having waist circumference ≥90 cm for males and ≥85 cm for females; (2) hyperglycemia, that is, having fasting blood glucose ≥6.1 mmol/L or 2-hour postprandial glucose ≥7.8 mmol/L, and/or those who have been diagnosed with hyperglycemia and in treatment; (3) hypertension, that is, those with blood pressure ≥130/85 mm Hg, and/or those who have been diagnosed with hypertension and in treatment; (4) fasting triglyceride ≥1.70 mmol/L; and (5) fasting high-density lipoprotein cholesterol <1.04 mmol/L
- Clearly understand the trial and voluntarily sign the informed consent form

**Exclusion criteria**

- Diagnosed with type 1 diabetes, steroid-induced diabetes, gestational diabetes, or specific types of diabetes
- Diabetes accompanied by severe complications such as diabetic nephropathy and diabetic ketoacidosis
- Secondary obesity (eg, secondary to pituitary inflammation, tumor)
- Secondary hypertension (eg, pheochromocytoma, renal hypertension)
- Secondary hyperlipidemia (eg, hypothyroidism, nephrotic syndrome)
- With severe heart, liver, or kidney disease or bleeding disorders, or with other serious diseases (eg, cancer, dementia)
- With severe mental disorders, speech, or hearing impairments
- Pregnant or lactating females
- Currently in another clinical trial

### Study Outcomes

The primary outcome will be the TCM syndrome differentiation in patients with T2DM and MetS, as assessed by the Syndrome Differentiation Questionnaire for T2DM and MetS (SDQTM). SDQTM is developed according to the Guideline for the Integrated Diagnosis and Treatment of Metabolic Syndrome [20], Guideline for the Integrated Diagnosis and Treatment of T2DM [21], and Guiding Principles for Clinical Research of New Chinese Medicine [22]. It includes 69 items (Textbox 2) that capture disease nature (eg, wind, dryness, yang deficiency) and location (eg, heart, kidney) [23]. Each item is a closed-ended question featuring a ranked scale with descriptors for symptom severity and frequency. Registered Chinese medicine practitioners will administer SDQTM in a face-to-face consultation integrating the 4 TCM diagnostic methods.

**Items of The Syndrome Differentiation Questionnaire for Type 2 Diabetes Mellitus and Metabolic Syndrome (SDQTM).**

1. Fatigue and weakness
2. Short of breath and want of speech
3. Spontaneous sweating
4. Pale or sallow yellow complexion
5. Pale lips and fingernails
6. Dizziness
7. Dry throat and mouth
8. Sensation of heat in the palms, soles, and chest
9. Hot flashes or night sweats
10. Aversion to cold and cold limbs
11. Puffy face and edema of the feet
12. Weak cough
13. Susceptibility to common colds
14. Reduced food intake and poor appetite
15. Abdominal distension after eating
16. Lower back and knee weakness
17. Tinnitus and deafness
18. Loose teeth and hair loss
19. Decreased libido
20. Palpitations
21. Insomnia and profuse dreaming
22. Forgetfulness
23. Blurred vision
24. Lusterless nails
25. Numbness in the hands and feet
26. Dry eyes
27. Irritability or depression with frequent sighing
28. Cold pain in the abdomen
29. Thirst with desire for cold drinks
30. Excessive eating and easy hunger
31. Fullness in chest and hypochondrium
32. Distending pain in the abdomen
33. Generalized edema
34. Heavy sensation in the limbs
35. Dry mouth and bitter taste
36. Dusky or dull dark complexion
37. Purplish dark lips
38. Squamous and dry skin
39. Fixed pain
40. Petechiae on the skin
41. Nausea
42. Vomiting
43. Itching skin
44. Sticky, greasy sensation in the mouth
45. Dark yellow or reddish urine
46. Frequent urination in large volume
47. Frequent nocturia
48. Scanty urine
49. Dry hard stools
50. Loose stools
51. Atonic constipation
52. Dark urine and constipation
53. Enlarged tongue with teeth marks
54. Pale tongue
55. Red tongue with scant coating
56. Pale, enlarged tongue with a moist coating
57. Red tongue with yellow coating
58. Yellow and greasy tongue coating
59. Purplish dark tongue, sublingual vein engorgement
60. Slippery and greasy tongue coating
61. Weak and forceless pulse
62. Thready and weak pulse
63. Thready and rapid pulse
64. Deep and slow pulse
65. Rapid pulse
66. Wiry pulse
67. Wiry and slippery pulse
68. Choppy or intermittent pulse
69. Slippery pulse

The secondary outcomes include demographic and anthropometric measurements, blood pressure, fasting glucose, glycosylated hemoglobin, lipid panel (total cholesterol, triglycerides, high-density lipoprotein, low-density lipoprotein), International Physical Activity Questionnaire-Short Form, Food Frequency Questionnaire, Pittsburgh Sleep Quality Index, Audit of Diabetes-Dependent Quality of Life, Framingham Stroke Risk Score (FSRS), retinal imaging, and concurrent medications.

Physical activity levels will be assessed using International Physical Activity Questionnaire-Short Form by recalling the time and days spent on 4 intensity levels (vigorous, moderate, walking, and sitting) over the previous 7 days [24]. Dietary intake will be assessed using a Food Frequency Questionnaire that is developed for older adults in Hong Kong and requires less than 30 minutes to complete [25]. The Pittsburgh Sleep Quality Index, a 19-item instrument, will be used to evaluate sleep quality over a 1-month interval, with its items generating 7 component scores that are summed into a global score ranging from 0 to 21, where higher scores indicate poorer sleep quality [26]. Audit of Diabetes-Dependent Quality of Life assesses diabetes-specific quality of life through 2 overview items and 19 items covering domains that include leisure, work, physical health, social relationships, and living conditions. A mean weighted impact score will be calculated, with lower scores indicating poorer diabetes-specific quality of life [27]. The FSRS will be used to estimate the individual risk of cardiovascular events, calculated based on age, smoking status, use of antihypertensive medications, blood pressure, diabetes status, high-density lipoprotein, and cholesterol levels [28]. Retinal microvasculature will be noninvasively visualized and quantified by fundus photography and computerized image analysis via a cloud-based artificial intelligence platform (Airdoc, Beijing Airdoc Technology Co, Ltd) to analyze its relation to cardiovascular events [29].

### Data Management and Monitoring

Participant confidentiality will be maintained using unique identifier codes, with de-identified data entered directly into the secure REDCap (Research Electronic Data Capture) database [30]. Electronic and hard-copy data will be stored on password-protected university servers and in locked cabinets, respectively, and retained for 7 years after the study. As this noninterventional study presents no anticipated risks, a data monitoring committee is not required, and monitoring will be performed by the principal investigator and the research team.

### Sample Size Calculation

The minimum required sample size is calculated using the single-population proportion formula [31], based on T2DM with MetS prevalence of approximately 11.2% in Hong Kong [32]. With a 95% confidence level, a precision of SD 3%, and an assumed nonresponse rate of 10%, the study aims to recruit 470 participants.

### Statistical Analysis

Statistical analysis will be performed using SPSS (version 29.0; IBM Corp), Python (version 3.13.9), and R (version 4.5.2; R Foundation for Statistical Computing) software. Missing values will be imputed by multiple imputation using the chained equation method. Quantitative data will be summarized as mean (SD) if normally distributed or as median and interquartile range otherwise. Latent class analysis will be used to identify the TCM syndrome patterns. Factor analysis will be used to reduce the dimensions of TCM syndrome elements and explore their underlying structure. Data suitability will be evaluated using the Kaiser-Meyer-Olkin measure and Bartlett's test of sphericity. Principal component analysis with varimax rotation will be employed to extract the common factors. Based on the factor analysis results, hierarchical cluster analysis will be performed to classify TCM syndrome elements. Cluster boundaries will be determined by identifying large distances between successive fusion levels in the dendrogram. The adjusted Rand index will be used for measuring the agreement between classifications.

For the comparison of quantitative data, one-way analysis of variance will be used if the assumptions of normality and homogeneity of variance are met. Otherwise, the Kruskal-Wallis test will be used. Categorical data will be expressed as numbers and percentages, and comparisons will be conducted using the chi-square test or Fisher exact test, as appropriate. Spearman correlation analysis will be employed to evaluate correlations between continuous variables, and Kendall’s tau-b analysis will be used for ordinal variables. Logistic regression will be performed to examine the relationship between syndrome differentiation and MetS components. A 2-sided value of *P*<.05 will be considered statistically significant.

## Results

This trial was registered on November 17, 2024. Participant recruitment for this study was initiated in November 2024. As of October 2025, more than 450 eligible participants have been enrolled and have completed data collection. Recruitment is scheduled to conclude on December 31, 2025.

## Discussion

### Overview

DM poses a critical global health challenge, with 65%-85% of individuals with T2DM also meeting the diagnostic criteria for MetS [5,6], underscoring a widespread profile of metabolic dysregulation. Given the high comorbidity, this large-scale cross-sectional study will be the first in Hong Kong to systematically characterize the distribution of TCM syndromes in this population. It will explore how TCM syndromes relate to important health-related behaviors such as physical activity, diet, and sleep quality, as well as quality of life. By clarifying the relationship between TCM syndromes and metabolic profiles, this study aims to combine TCM assessments with clinical indicators, support more tailored treatments, and underscore the value of TCM syndrome differentiation in managing and predicting outcomes in patients with T2DM and MetS.

### Anticipated Principal Findings

Although TCM syndrome distributions in patients with T2DM and MetS have been previously examined separately [16,33,34], this study specifically focuses on their comorbidity. We anticipate that the TCM syndrome profile of T2DM-MetS comorbidity will demonstrate a distinct distribution pattern compared to either condition alone, exhibiting unique relationships with disease duration and severity. Given that both conditions represent independent cardiovascular risk factors, we further hypothesize that specific TCM syndrome patterns will correlate with particular metabolic abnormalities and varying risks of disease progression. Through the combined application of FSRS and retinal imaging, we will be able to quantitatively characterize cardiovascular risks across different syndrome patterns. Furthermore, we expect these TCM syndromes to be influenced by modifiable health behaviors and associated with differences in the quality of life. Collectively, these findings will provide evidence supporting the clinical value of TCM syndrome differentiation in prognostic stratification and personalized management of T2DM and MetS.

### Strengths and Limitations

This study has several strengths, including its design as the first large-scale cross-sectional investigation systematically characterizing TCM syndromes in individuals with T2DM and MetS. This research innovatively integrates clinical indicators such as biochemical profiling and retinal imaging with TCM syndrome differentiation while also incorporating evaluations of health behaviors and quality of life. However, several limitations should be acknowledged. The cross-sectional design inherently prevents establishing causal relationships, and reliance on self-reported data may introduce recall bias. Additionally, potential confounding factors could affect result interpretation, and the generalizability of the findings beyond the studied population requires further validation.

### Implications for Practice or Research

The findings may provide practitioners with a practical framework for incorporating TCM syndrome differentiation into T2DM and MetS management. By identifying syndrome patterns associated with metabolic profiles and lifestyle factors, this approach supports personalized treatment strategies that combine biomedical and traditional diagnostic perspectives. The integration of retinal imaging and cardiovascular risk assessments with TCM diagnostics further provides a model for comprehensive evaluation in integrative medicine practice.

Beyond these clinical implications, this study underscores the need for validated TCM syndrome assessment tools to standardize future research. It also calls for longitudinal research to establish causal relationships between syndrome patterns and disease progression. Further investigation is warranted to explore the biological mechanisms underlying identified syndrome configurations, potentially through multi-omics approaches [35]. Intervention trials targeting specific syndrome patterns would advance evidence for personalized TCM therapies.

### Conclusion

This study will first systematically characterize TCM syndromes in patients with T2DM and MetS by integrating clinical indicators with traditional diagnostic approaches. The findings are expected to reveal significant relationships between syndrome differentiation, metabolic parameters, lifestyle factors, and disease progression. This work may establish a framework for incorporating TCM syndrome differentiation into chronic disease management, thereby contributing to the development of personalized treatment strategies and improved patient outcomes in integrative medicine practice.

### Dissemination

Results will be disseminated through peer-reviewed journals and presentations at international conferences focused on metabolic disorders, ensuring accessibility to clinicians, researchers, and policymakers.

## Data Availability

The datasets generated or analyzed for this study are available from the corresponding author on reasonable request.

## Appendix

Multimedia Appendix 1 STROBE checklist.

## Abbreviations

DM: diabetes mellitus
FSRS: Framingham Stroke Risk Score
MetS: metabolic syndrome
REDCap: Research Electronic Data Capture
SDQTM: Syndrome Differentiation Questionnaire for Type 2 Diabetes Mellitus and Metabolic Syndrome
STROBE: Strengthening the Reporting of Observational Studies in Epidemiology
T2DM: type 2 diabetes mellitus
TCM: Traditional Chinese Medicine
